# Medical Association in Lake and MeHenry Counties Illinois

**Published:** 1847-09

**Authors:** 


*Medical Association in Lake and McHenry Counties Illinois.*—We have received a number of the *Lake County Visiter*, published at Little Fort, Illinois, containing one of a series of articles on the formation of a Medical Society in that and the adjoining county of McHenry. Appended to the article, which is written with ability, are initials which we recognize as those of the name of a young friend of much promise, who we are glad to see is engaging with zeal in matters relating to the interests of our common profession. With our sentiments on the importance of associations our readers are sufficiently acquainted. We regard them as the breath of the life of medical progress, and we trust that the exertions of our friend will be crowned with success.

